# Early Coronary Artery Calcification Progression over Two Years in Breast Cancer Patients Treated with Radiation Therapy: Association with Cardiac Exposure (BACCARAT Study)

**DOI:** 10.3390/cancers14235724

**Published:** 2022-11-22

**Authors:** Manoj Kumar Honaryar, Rodrigue Allodji, Jean Ferrières, Loïc Panh, Médéa Locquet, Gaelle Jimenez, Matthieu Lapeyre, Jérémy Camilleri, David Broggio, Florent de Vathaire, Sophie Jacob

**Affiliations:** 1 INSERM U 1018, CESP, Radiation Epidemiology Team, 94800 Villejuif, France; 2 Institute Gustave Roussy, 94800 Villejuif, France; 3 University Paris-Saclay, 94800 Villejuif, France; 4 Department of Cardiology and INSERM UMR 1295, Rangueil University Hospital, 31400 Toulouse, France; 5 Department of Cardiology, Clinique Pasteur, 31076 Toulouse, France; 6 Laboratory of Epidemiology, Institute for Radiation Protection and Nuclear Safety (IRSN), 92260 Fontenay-Aux-Roses, France; 7 Department of Radiation Oncology (Oncorad), Clinique Pasteur, 31076 Toulouse, France; 8 Department of Radiology (GRX), Clinique Pasteur, 31076 Toulouse, France; 9 Department of Dosimetry, Institute for Radiation Protection and Nuclear Safety (IRSN), 92260 Fontenay-Aux-Roses, France

**Keywords:** breast cancer, radiation therapy, coronary artery calcification, cardiotoxicity, dosimetry

## Abstract

**Simple Summary:**

Radiotherapy for breast cancer can induce radiation-induced coronary artery diseases many years after RT. Long before the onset of clinical signs of these diseases, increase in coronary artery calcium (CAC) score is a good predictor of the risk of coronary disease in patients who may be asymptomatic. In order to improve knowledge for the prevention of radiation-induced coronary artery diseases, our study aimed to evaluate whether there was an association between early CAC increase and cardiac exposure. Based on a population of 101 breast cancer patients with CAC score measured before and 2 years after RT, CAC increase was observed in 28 patients presenting higher cardiac exposure than others. Calcifications were mainly localized in the left anterior descending coronary, the most exposed coronary artery. This study suggests that minimizing cardiac exposure could limit the risk of early CAC increase and therefore the longer-term risk of coronary artery diseases.

**Abstract:**

Background: Radiotherapy (RT) for breast cancer (BC) can induce coronary artery disease many years after RT. At an earlier stage, during the first two years after RT, we aimed to evaluate the occurrence of increased coronary artery calcium (CAC) and its association with cardiac exposure. Methods: This prospective study included 101 BC patients treated with RT without chemotherapy. Based on CAC CT scans performed before and two years after RT, the event ‘CAC progression’ was defined by an increase in overall CAC score (CAC RT+ two years—CAC before RT > 0). Dosimetry was evaluated for whole heart, left ventricle (LV), and coronary arteries. Multivariable logistic regression models were used to assess association with doses. Results: Two years after RT, 28 patients presented the event ‘CAC progression’, explained in 93% of cases by a higher CAC score in the left anterior descending coronary (LAD). A dose–response relationship was observed with LV exposure (for Dmean LV: OR = 1.15, *p* = 0.04). LAD exposure marginally explained increased CAC in the LAD (for D2 LV: OR =1.03, *p* = 0.07). Conclusion: The risk of early CAC progression may be associated with LV exposure. This progression might primarily be a consequence of CAC increase in the LAD and its exposure.

## 1. Introduction

Breast cancer (BC) is the most common cancer in women, with more than two million new cases and almost 685,000 deaths in 2020 worldwide [1]. Despite the beneficial effects of radiotherapy (RT) in reducing loco-regional recurrence and mortality of BC patients [2], it has been shown that RT can induce adverse cardiovascular events and an excessive mortality from cardiac diseases [3,4,5].

Among these cardiovascular complications, coronary artery disease (CAD) is the most common manifestation of radiation-induced heart disease [3], and it has been demonstrated that RT for BC is associated with an increased risk of CAD, which is proportional to the mean heart dose (7.4% to 16.5% per additional Gy) [6,7]. In addition, such complications are more commonly seen in patients with left-sided rather than right-sided BC as a larger portion of the heart [6]. In particular, the left anterior descending coronary artery (LAD) is included in the radiation field. 

Baseline measures of coronary artery calcification (CAC), as quantified by cardiac computed tomography (CT), have been shown to predict future cardiovascular events, in particular CAD, in multiple populations [8]. In the population of BC patients treated with RT, several studies found that pre-treatment CAC was associated with acute coronary event and CAD risk [9,10,11].

Although baseline CAC might reflect prior coronary atherosclerotic plaque burden, CAC progression might provide insight into current disease activity [12]. The impact of heart irradiation in BC patients on CAC progression was poorly investigated. A study showed that CAC change evaluated seven years after BC RT was less increased in left-sided BC patients with breath-hold technique (allowing lower cardiac exposure) compared to left-sided BC patients without breath-hold [13], and a higher risk of accelerated CAC burden was found in the left-sided BC patients compared to right-sided BC patients after adjuvant RT [14]. However, no precise evaluation of dose–response relationship was investigated, and little is known on early CAC progression occurring within two years after RT and its potential association with unavoidable cardiac radiation exposure.

Based on the BACCARAT study (BreAst Cancer and CArdiotoxicity Induced by RAdioTherapy) [15], a prospective cohort which included BC patients treated with 3D Conformal Radition Therapy (CRT) followed for two years, the aim of this study was to assess the occurrence of CAC progression based on measurements of CAC before and two years after RT and to evaluate whether cardiac exposure, determined to heart, left ventricle, and coronary arteries doses, was associated with early progression of CAC.

## 2. Materials and Methods

### 2.1. Study Design and Population

The BACCARAT study is a monocentric prospective cohort of 118 female volunteer patients treated for BC from 2015 to 2017 in the Clinique Pasteur, Toulouse, France [15]. All patients were treated with adjuvant 3D-CRT after surgical treatment (conservative or mastectomy) without chemotherapy. The age range at the time of BC treatment was 40 to 75 years. In order to have a large range of heart doses, which is useful for analysis of dose–response relationships of cardiotoxicity, we prospectively included patients with left BC (85%), but also a smaller proportion of right BC (15%) with lower heart doses. Deep inspiration breath hold (DIBH) was only used for patients treated for left BC with heart very close to the anterior chest wall or for dose constraints achievement according to Clinic Pasteur practices (mean heart dose < 5Gy and V25Gy < 10 %). Patient medical history was collected, and physical examinations were performed by the cardiologists during consultation. Patients were followed for two years, including cardiac imaging examination, in particular cardiac CT performed at baseline before RT and two years after RT. Five patients withdrew consent and twelve patients had missing data (either dosimetry or CAC measurements). Finally, the study population presented here consisted of 101 patients with complete CAC score data at baseline and RT+ two years follow-up, as well as dosimetry data. 

This study received ethical approval from the French South West Committee for Protection of Persons (ID: CPP2015/66/2015-A00990-69) and from the National Agency for Medical and Health products Safety (Reference: 150873B-12). All patients enrolled in the study provided their written informed consent.

### 2.2. Radiotherapy 

At first, all the BC patients underwent surgical treatment (mastectomy or lumpectomy). Afterwards, patients were treated with 3D-CRT with or without irradiation of supraclavicular or internal mammary lymph nodes. Patients were positioned on a breast board with both arms above the head. The planning target volume dose was 50 Gy delivered in 5 weeks with 25 daily doses of 2 Gy or 47 Gy delivered in 5 weeks with 20 daily doses of 2.35 Gy over 5 weeks for patients treated between January 2016 and May 2016. This hypo-fractioned dose administration decision was made due to technical problems in one of the 3D-CRT machines during this period and due to the need to slightly limit the number of sessions per patient. Six MV photons were used for most of the patients, with the exception of the few cases of patients with big breast sizes where 25 MV additional photons were delivered. An additional boost of 9 to 15 Gy could be applied to the tumor site with electron/photons beams, with energies ranging from 6MeV to 18 MeV on a case-by-case basis. Eclipse™ Treatment planning system (TPS) and the integrated software Analytical Anisotropic Algorithm (AAA v13.6) (Varian Medical System, Palo Alto, CA, USA) were used to conduct whole heart dose calculations. The resulting doses of all irradiated volumes were considered. The RT was planned for each patient so that the distribution was normalized and optimized based on the International Commission on Radiation Units and Measurements (ICRU) point of reference for the breast and to obtain QUANTEC dose constraints to organs at risk, e.g., the heart [16]. 

### 2.3. Radiation Doses 

The methods used for the evaluation of radiation doses distribution to the whole heart, left ventricle, left main coronary artery (LMCA), left anterior descending artery (LAD), circumflex artery (Cx), and right coronary artery (RCA) in BACCARAT patients are described elsewhere [15,17]. In summary, the RT department of the Clinic Pasteur produced the Dose–Volume Histogram (DVH) for the heart. The delineation of the cardiac sub-structures was performed manually. DVHs for additional cardiac sub-structures were generated with ISOGray TPS by the dosimetry department of IRSN in collaboration with the Clinic Pasteur RT department by using the 3D dose matrix created during planning treatment. From the DVHs, the following absorbed dose metrics for cardiac structures and coronary arteries were calculated: Dmean (in Gy) is the volume-weighted mean dose; D2 (in Gy) is the minimal dose received by the highly irradiated 2% of the structure volume, which can be considered as close to maximum dose; V2 (in %) is the relative volume of the concerned structure exposed to at least 2 Gy, V5 (in %) exposed to 5 Gy.

### 2.4. CAC CT Scans

The CAC CT scans were carried out before treatment and two years after RT. CAC was measured using ECG-Gated Ct exams without contrast injection acquired on a SIEMENS dual-source CT (SOMATOM FLASH definition, Siemens Imaging system ®, Erlangen, Germany). The noninvasive CT scan was performed within 10–15 min, with a 3-mm reconstructed slice thickness. A calcified lesion was defined as more than three contiguous pixels with a peak of attenuation of at least 130 Hounsfield units (HU). The overall CAC score was calculated according to Agatston et al. [18], using a Siemens Syngovia Workstation, by summing individual lesion scores from each of the main epicardial coronary arteries: LMCA, LAD, LCX, and RCA. All CAC scores were evaluated by one experimented radiologist (ML, 15 years of experience in cardiovascular imaging) blinded for the side of the RT.

### 2.5. Endpoint CAC Progression

For all patients, we had measurements of the overall CAC and CAC in each coronary artery before RT and two years after RT. Several definitions exist for quantification of the progression of the CAC scores [19,20], and there is no consensus for definition and cut-off values depending on the population and the later endpoint considered (general population/patients; incidence/mortality, etc.). With a follow-up of two years in our study, we wanted to detect early CAC progression, possibly at the stage of subclinical changes. In order to be as sensitive as possible, we thus defined our endpoint CAC progression (yes/no) as an increase in overall CAC score: CAC RT+ two years—CAC before RT > 0. 

### 2.6. Statistical Analyses 

The descriptive analyses were expressed as means and standard deviations (SD), medians and interquartile ranges (IQR) for quantitative variables, absolute numbers (n), and relative percentages (%) frequencies for qualitative variables. The comparisons were performed using the non-parametric Wilcoxon test for continuous data (dosimetry data 3 and CAC scores) and Mac Nemar or Chi2 for categorical data (proportions and percentages). We analyzed the associations between the endpoint CAC progression (yes/no) and radiation and non-radiation factors in univariate analysis based on logistic regressions (odds ratios (ORs), 95% confidence intervals (CI), p-values). Cardiac radiation exposure factors included the laterality of BC, Dmean, D2 and V2 and V5 of the heart, the left ventricle, and coronary arteries. Non-radiation factors included covariates known to have a potential impact on coronary atherosclerosis development and progression such as age, body mass index (BMI), smoking, hypertension, diabetes, dyslipidemia, endocrine therapy, cardiovascular treatment (including statins use), and baseline CAC score (zero/non-zero) [21]. Because of the limited size of the study and the important number of covariates, for parsimony purposes, in multivariable analysis of radiation exposure we only performed adjustment on non-radiation variables with *p*-value < 0.05 in univariate analysis. The analyses of the data were conducted with Stata 14.2 STATA corp. *p*-values < 0.05 (two-sided) were considered statistically significant.

## 3. Results

### 3.1. Characteristics of The Study Population 

Overall, 101 BC patients (84 left-sided and 17 right-sided) were included in this analysis. Baseline patients’ characteristics are shown in Table 1. The mean age at inclusion was 58.4 ± 8.1 years. The mean body mass index (BMI) was 24.4 ± 4.1 kg/m². Most patients were diagnosed with invasive cancer (81%), and experienced conservative treatment of the breast as surgical choice of treatment (94%) and 76% of patients received hormonal therapy. For about 75% of the patients, the standard protocol of RT of 50 Gy was prescribed, and only 25% benefited from a hypo-fractioned dose of 47 Gy. Concerning cardiovascular risk factors, 7% of patients had diabetes, 15% had hypertension, almost half of the patients were current or former smokers (45%), and 15% of patients had cardiovascular treatment at baseline (including 7% with statins) There were no significant differences in terms of baseline cardiovascular risk factors, cancer, and treatment characteristics among left-sided and right-sided BC patients.

### 3.2. Cardiac Doses

Detailed information on cardiac doses is provided in Table 2. The mean heart dose and left ventricle dose were 2.87 ± 1.28 Gy and 6.18 ± 3.24 Gy, respectively, for left-sided BC patients and 0.61 ± 0.46 Gy and 0.17 ± 0.29 Gy, respectively, for the right-sided BC patients. Doses were much lower for the right-sided BC patients versus left-sided BC patients (*p* < 0.001), except for the RCA dose, which was higher in patients treated for right BC compared to the left one. The LAD was the most exposed coronary artery with a mean dose of 16.0 Gy for left-sided BC. 

### 3.3. CAC Progression

The CAC values evaluated at baseline and two years after RT are described in Table 3. Before RT, 76 (75.3%) patients had zero CAC value and 25 (24.7%) patients had non-zero CAC value. Two years after RT, the proportion of non-zero CAC patients was slightly increased from 24.7% to 29.7% (*p* = 0.06) and 28 patients (27.7%) had CAC progression: 5 patients had zero baseline CAC value (5/76, 7%) and 23 patients had non-zero baseline value CAC (23/25, 92%), *p* < 0.001. A significant increase in mean overall CAC was observed (35.8 vs. 47.7, *p* < 0.001). Among 28 patients with CAC progression, 89% of patients had increased CAC in LAD (7% of patients had increased CAC in LMCA; 36% of patients had increased CAC in CX; 54% of patients had increased CAC in RCA), and the proportion of non-zero values for CAC in LAD significantly increased from 19.8% to 25.7% (*p* = 0.03), in contrast with other coronary arteries.

### 3.4. Association between CAC Progression and Non-radiation Cardiovascular Risk Factors 

Based on univariate logistic regression analysis, we evaluated the association between the occurrence of CAC progression and baseline cardiovascular risk factors (intrinsic factor and endocrine therapy) (Table 4). Patients with baseline CAC > 0 had a very high risk of CAC progression compared to patients with baseline CAC = 0 (92% vs. 7%, *p* < 0.00001). This baseline CAC status was thus a major risk factor of CAC progression that needed to be taken into account for further multivariable dose–response analysis. Moreover, age, hypertension, diabetes, and use of cardiovascular treatment reached statistical significance (*p* < 0.05) and were considered in multivariable analysis.

### 3.5. Association between CAC Progression and Cardiac Exposure

**Figure 1:**
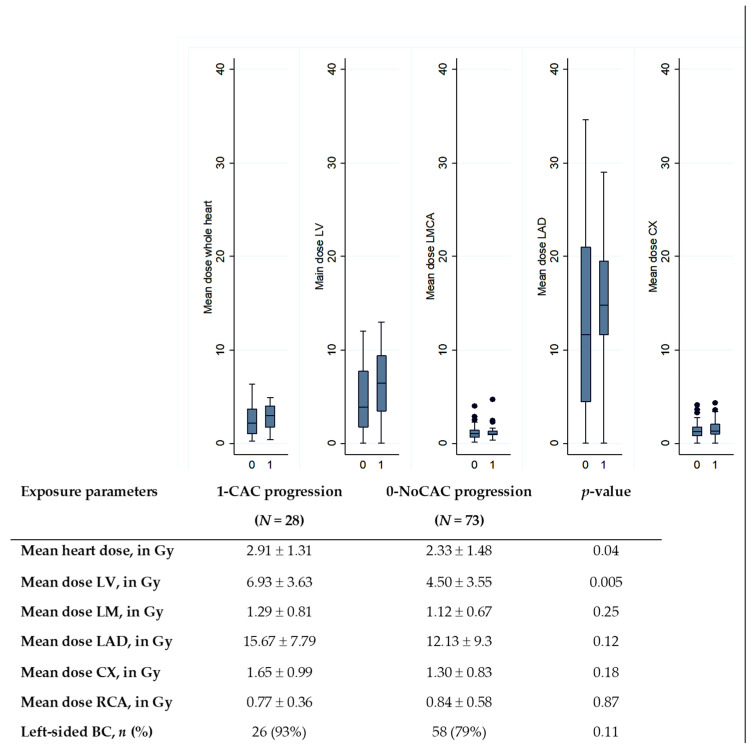
Comparison of cardiac doses according to the CAC progression status (0 for no CAC progression; 1 for CAC progression). LMCA: left main common coronary artery, LAD: left anterior descending artery, CX: circumflex artery; RCA: right coronary artery. The central value of the box indicates the median, the borders of the box indicate the quartiles (25th and 75th), and the extremities indicate the minimum and maximum values. *p*-value: results of Wilcoxon test to compare dose distributions.

A comparison of doses according to the CAC progression status is presented in Figure 1. We observed that mean heart dose and mean LV dose were significantly higher in the group of patients with CAC progression compared to patients without CAC progression (2.33 Gy vs. 2.91 Gy, *p* = 0.04 for heart; 4.50 Gy vs. 6.93 Gy, *p* = 0.005 for LV). No other cardiac substructure doses differed significantly between both groups. 

Results on the association between CAC progression and cardiac exposure are detailed in Table 5. Regarding laterality, left-sided BC patients presented a higher risk of CAC progression than right-sided BC, but the result was not significant (OR = 3.36, *p* = 0.12). We focused dose–response analysis on cardiac structures previously identified with significant difference in mean dose distribution (Figure 1): heart and left ventricle. In univariate analysis, no significant association between CAC progression and heart exposure was observed (*p* > 0.05), except D2 of the heart (OR = 1.03, *p* = 0.018). Several left ventricle exposure parameters were significantly associated with CAC progression, in particular mean LV dose, D2, V2, and V5. Given the limited size of the study and the strength of the association between baseline CAC and CAC progression (indicated in Table 4), in order to prevent over-adjustment, multivariate analyses for radiation doses were presented with two models: either adjusted on other non-radiation factors previously identified with *p* < 0.05 (Model 1 adjusted for age, hypertension, diabetes, cardiovascular treatment), or adjusted on baseline CAC status (zero/non-zero) and age (Model 2). In Model 1, we observed that the risk of CAC progression was associated with the mean LV dose with an increased risk of 15% per additional Gy (OR = 1.15, *p* = 0.043) and the risk increased by 4% per additional Gy in the near maximum dose D2 (OR = 1.04, *p* = 0.02). In Model 2, similar findings were observed with strengthened associations. 

**Figure 2:**
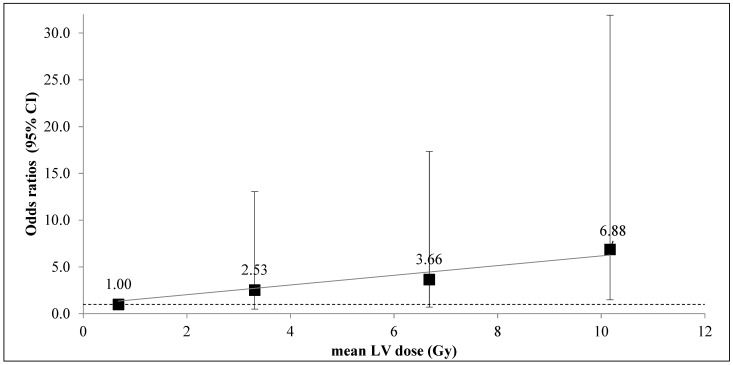
Association between CAC progression and mean LV dose in 4 categories: <1.79 Gy (reference category mean dose = 0.68 Gy, 26 patients, 3 patients with the event); 1.79 Gy–4.56 Gy (mean dose = 3.31 Gy, 25 patients, 5 patients with the event); 4.56 Gy–8.21 Gy (mean dose = 10.17Gy, 25 patients, 8 patients with the event); >8.21 Gy (mean dose = 10.17 Gy, 25 patients, 12 patients with the event). Odds ratios are adjusted for age, hypertension, cardiovascular treatment, and diabetes. For the highest category of dose, OR = 6.88 (1.48 – 31.91), *p* = 0.03. The grey line corresponds to the linear trend.

The analysis of CAC progression according to the mean LV dose category (divided in 25th percentile of mean dose distribution) (Figure 2), which allowed for the observation that patients in the highest mean LV dose category (>8.21 Gy) had a risk of CAC progression multiplied by nearly 7 compared to the low exposure category (<1.79 Gy), with a Model 1 adjusted OR = 6.88, *p* = 0.029.

### 3.6. Association between CAC Increase in the LAD and Cardiac Exposure

As previously indicated, CAC in the LAD was increased in 89% of patients with CAC progression. We further investigated the association between these localized increased CAC in LAD and dose to the heart, the left ventricle, and the LAD (Table 6). We could observe that the left ventricle exposure was significantly associated with increased CAC in LAD (OR = 1.20, *p* = 0.006 for mean LV dose; OR = 1.04, *p* = 0.008 for V2 of LV) in univariate analysis, and the association with V2 remained significant even after adjustment on age, diabetes hypertension, and cardiovascular treatment use (OR = 1.03, *p* = 0.037). Regarding the LAD exposure, only the near maximum dose was significantly associated with increased CAC in LAD (OR = 1.04, *p* = 0.02), but this association was only marginally significant (*p* = 0.07) in multivariate analysis.

## 4. Discussion

In this prospective study of 101 BC patients treated with 3D-CRT without chemotherapy, 28 patients had CAC progression characterized by an increase > 0 in CAC value from baseline to RT+ two years measurements. Non-zero CAC at baseline was a major risk factor of CAC progression, as well as age, hypertension, diabetes, and use of cardiovascular treatment (including statins). We observed a significant dose–response relationship between the risk of CAC progression and mean or near maximum LV dose, which remained significant even after adjustment on these intrinsic cardiovascular risk factors. This progression might primarily be a consequence of CAC increase in the LAD and its exposure. 

Both cardiac exposure and pre-treatment CAC, in BC patients treated with RT, have been shown to be associated with the risk of coronary artery disease several years after RT [6,7,9,10,11]. At an earlier stage of the coronary artery disease, assessment of CAC progression is regarded as a dynamic measurement that might provide insight into ongoing current disease activity and more efficiently predicts future cardiac events rather than static traditional clinical parameters and baseline CAC [12]. Several methods exist for quantification of the progression of the CAC scores that are based, among others, on absolute difference between the second and first measure of CAC or the percent change [19]. However, there is no consensus for definition and cut-off values for CAC progression as it depends on the endpoint investigated (incidence, mortality) or the type of population (cardiac patients, other patients, general population from several countries). Some studies based on cardiac patients reported annual CAC progression rates based on percent change from 10% to 30 % [19,22,23]. In our study, we were interested in early progression of CAC arising after two years RT in BC patients with no history of coronary artery disease. For this purpose, we wanted to be very sensitive in the detection of CAC progression, possibly at the stage of subclinical changes, and we considered CAC progression for patients with any increase from baseline to RT+ two years measurement. Based on this definition, 28% of our population had CAC progression, but this result is difficult to compare with other studies in the absence of guidelines for definition CAC progression. Several approaches were used in studies on patients with cancer treated with RT. A study showed that three years after BC RT, the CAC score was less increased compared to baseline value, and in left-sided BC patients with breath-hold technique (allowing lower cardiac exposure) compared to left-sided BC patients without breath-hold [13]. Similarly, we also found in our study a significant increase in mean overall CAC from baseline to RT+ two years among left-sided BC but not in right-sided BC (Table 3). A study performed in Taiwan [14] compared BC patients treated with RT with non-BC women as a control group by using changes in CAC percentile (%CACinc = CAC percentile > 1 year after RT−CAC percentile before RT). CAC progression was defined with a cut-off of more than 50 percentile and this study showed that the risk of CAC progression was higher in left-sided BC compared to right-sided BC patients. With a different definition of CAC progression, our study also showed a higher frequency of CAC progression in left-sided BC compared to right-sided BC (11.7% vs. 30.1%, OR = 3.2, *p* = 0.16). However, some other studies did not observe significant difference in CAC before and after RT, which may be explained by a short follow-up (< 1 year) [24,25].

Among these previous studies, none provided a precise evaluation of the dose–response relationship between cardiac exposure and CAC progression, which is the best approach to enhance knowledge on causality association between exposure and radiation-induced cardiac endpoint. Our study was the first to present results on CAC progression evaluated two years after RT and combined with a precise evaluation of cardiac exposure, including left ventricle and coronary arteries. A dose–response relationship between mean LV dose and the occurrence of CAC progression was observed, and this result is concordant with previous study, indicating a dose–response relationship between LV exposure and the occurrence of acute coronary event [7]. Interestingly, our association with cardiac doses (in particular, LV doses) remained significant even after adjustment on baseline intrinsic risk factors of atherosclerosis, which may have fully explained CAC progression. In particular, it is known that elevated CAC scores are predictive of future cardiac events [26] and baseline CAC status (zero/non-zero) was shown to be a powerful predictor of CAC progression. Gopal et al. [27] showed that only 2% of patients with zero baseline CAC had CAC progression > 50 within five years of follow-up. In our study, we found that only 7% of patients with zero baseline CAC had CAC progression >0 two years after RT (92% for patient with non-zero baseline CAC), which is quite consistent with Gopal et al.’s results. Simonetto et al. [28], based on a process-oriented model of acute coronary event, also illustrated the major contribution of baseline CAC or age in the risk of advanced atherosclerosis after RT, and mean heart dose remained associated with the acute coronary event risk only in cases of already existing calcified plaques. This raises the question of whether cardiac radiation exposure after RT would be involved in initiation or progression of atherosclerosis. Despite low statistical power, in our group of 76 patients without existing calcified plaques (i.e., with zero baseline CAC), a positive non-significant association with mean LV dose could be observed (OR = 1.2 (0.9–1.6), *p* = 0.11), which may illustrate that radiation exposure could also be involved in the initiation process. This remains to be further investigated.

We observed that in nearly all cases of CAC progression after RT, calcifications were localized in the LAD. In BC RT context, this is not surprising for several reasons. First, the LAD is the most exposed coronary artery that can receive hot spot doses > 40 Gy [17] and may result in atherosclerosis after RT. Second, as suggested by Nilsson et al. [29], incident plaques or progression of plaques were most frequently observed in the LAD compared to other coronaries. Our study could not find a strong association between LAD exposure and increase of CAC in LAD but suggests further investigating this point, as the near-maximum dose of LAD may be an important parameter to consider.

Some studies proposed cut-off values to define CAC progression as a strong predictor of coronary event or mortality [19], but little is known on the clinical implications of decreasing CAC progression, and it is unclear whether decreased CAC progression could be achieved with currently available medications. With a cut-off value of 0 in CAC change, which involved a very sensitive definition of CAC progression, our study suggested that in the context of heart irradiation of BC patients treated with RT, for primary prevention, limitations in cardiac doses, in particular left ventricle doses, might be an interesting measure to prevent the risk of CAC progression. More prospective data are needed to further elucidate whether quantifying early CAC progression can be recommended for cardiac risk stratification in clinical practice for BC patients treated with RT or if it can be used as an early or mid-term surrogate for clinical end points in future trials.

### Limitations

This study has several limitations. Our study was relatively small, but it is one of the largest ever published on CAC progression in BC RT population, and we could observe a statically significant dose–response relationship. Our definition of endpoint CAC progression was based on very sensitive thresholds (>0) for arithmetical difference in CAC scores at baseline and RT+ two years, which probably resulted in overestimation of clinically significant progression of CAC, but this may have involved underestimation of OR. As a consequence, the significant association observed with LV exposure can be considered as relevant. We included patients having received RT without chemotherapy, allowing us to specifically analyze radiation-induced CAC progression without potential bias due to combination with chemotherapy. A large multicenter European study (MEDIRAD EARLY-HEART study) is ongoing and included 250 patients, which should provide results without this size limitation [30,31]. With limited sample sizes, we could not investigate whether the association between CAC progression and cardiac exposure could be observed in both patients with zero and non-zero baseline CAC. This would help to clarify whether cardiac exposure is involved in either initiation or progression or both and would be of interest to enhance knowledge on the impact of RT on initiation or development of atherosclerosis surrogate. 

Our study focused on CAC CT Scan and CAC scores. However, the absence of measured CAC (CAC = 0) does not absolutely rule out the absence of atherosclerotic plaques, including other forms of non-calcified plaques such as unstable plaques and soft plaques. With a zero CAC, there is, however, a low probability of significant luminal occlusion [12]. Further investigation of non-calcified plaques will be possible for patients included in BACCARAT who also had, at baseline and two years after RT, a contrast injected CTCA, just after the non-contrast CAC CT [15]. Data regarding theses examination are being collected and analyzed and should complete knowledge on both calcified and non-calcified atherosclerotic plaques arising with two years after RT. 

## 5. Conclusions

The study found an association between LV exposure and the risk of CAC progression within two years after BC RT, even after adjustment on intrinsic cardiovascular risk factors. Whether such a progression is mainly the consequence of CAC increase in the LAD and its exposure is suggested but remains to be confirmed. These results prompt further investigation of the early- and mid-term development of radiation-induced calcifications and their later consequences.

## Figures and Tables

**Table 1:** Baseline characteristics of the study population.

Variables	Patients *N* = 101
Age in years, mean ± SD	58.4 ± 8.1
**Cancer treatment**	
Laterality of BC, *n* (%) Left-sided BC Right-sided BC	84 (83.2%) 17 (16.8%)
Histology, *n* (%) In situ Invasive	19 (18.8%) 82 (81.2%)
Cancer grade, *n* (%) 1 2 3	39 (38.6 %) 50 (49.5%) 12 (11.9%)
Type of surgery, *n* (%) Conservative Mastectomy	94 (93.1%) 7 (6.9%)
Protocol of radiotherapy, *n* (%) 50 Gy (25 x 2Gy) 47 Gy (20 x 2.35Gy)	76 (75.2%) 25 (24.8%)
Regional lymph nodes irradiation, *n* (%) No Yes (Supraclavicular and/or Internal mammary Chain)	73 (72.3%) 28 (27.7%)
Boost, *n* (%) No Yes	9 (8.9%) 92 (91.1%)
Adjuvant endocrine therapy, n (%) No Yes Anti-aromatase * Tamoxifen *	24 (23.8%) 77 (76.2%) 45 32
**Cardiovascular risk factors**	
Body mass index in kg/m², mean ± SD	24.4 ± 4.1
Cardiovascular treatment, *n* (%)	15 (14.8)
Diabetes mellitus, *n* (%)	7 (6.9%)
Hypertension, *n* (%)	15 (14.8%)
Smoking status, *n* (%) No Former Current	55 (54.4%) 24 (23.7%) 22 (21.7%)

* Type of endocrine therapy.

**Table 2:** Description of cardiac mean doses and dose–volume histogram parameters.

Dosimetric Variables	All Patients *N* = 101	Right-Sided BC *N* = 17	Left-Sided BC *N* = 84	Comparison Right vs. Left
	Mean ± SD	Mean ± SD	Mean ± SD	*p*-Value ^1^
**Whole heart** Mean dose (Gy) D2(Gy) V2(%) V5 (%)	2.49 ± 1.45 22.67 ± 17.85 24.0 ± 14.9 7.6 ± 6.4	0.61 ± 0.46 2.47 ± 1.14 6.7 ± 13.0 0.3 ± 1.0	2.87 ± 1.28 26.76 ± 16.83 27.4 ± 12.7 9.1 ± 6.0	<0.001 <0.001 <0.001 <0.001
**Left ventricle** Mean dose (Gy) D2(Gy) V2(%) V5 (%)	5.17 ± 3.72 28.62 ± 18.93 40.4 ±22.2 18.2 ± 12.5	0.17 ± 0.29 0.54 ± 0.60 1.0 ± 4.3 0 ± 0	6.18 ± 3.24 34.30 ± 15.40 48.4 ± 14.5 21.9 ± 10.3	<0.001 <0.001 <0.001 -
**LMCA** Mean dose (Gy) D2(Gy) V2(%) V5 (%)	1.17 ± 0.71 1.48 ± 0.91 8.9 ± 25.1 0 ± 0	0.63 ± 0.76 0.69 ± 0.80 8.3 ± 23.4 0 ± 0	1.27 ± 0.66 1.64 ± 0.84 11.7 ± 33.2 0 ± 0	<0.001 <0.001 0.431 -
**LAD** Mean dose (Gy) D2(Gy) V2(%) V5 (%)	13.11 ± 9.02 32.13 ± 19.25 71.9 ± 33.3 46.7 ± 28.1	0.28 ± 0.53 0.51 ± 0.74 6.6 ± 22.2 0 ± 0	15.71 ± 7.57 38.53 ± 14.14 85.1 ± 13.8 56.2 ± 20.3	<0.001 <0.001 <0.001 -
**CX** Mean dose (Gy) D2(Gy) V2(%) V5 (%)	1.40 ± 0.89 2.36 ± 4.79 18.3 ± 30.2 0.5 ± 4.6	0.28 ± 0.43 0.53 ± 0.72 2.9 ± 8.2 0 ± 0	1.63 ± 0.78 2.73 ± 5.16 21.4 ± 32.0 0.6 ± 5.1	<0.001 <0.001 <0.001 -
**RCA** Mean dose (Gy) D2(Gy) V2(%) V5 (%)	0.82 ± 0.53 1.42 ± 0.84 5.0 ± 15.1 0.14 ± 1.48	1.46 ± 0.78 2.10 ± 1.23 17.5 ± 27.0 0.87 ± 3.60	0.69 ± 0.34 1.28 ± 0.67 2.4 ± 9.8 0 ± 0	<0.001 <0.001 <0.001 -

BC: breast cancer, SD: standard deviation, D2: (Gy) minimal dose received by the most irradiated 2% of structure volume, V2–V5 (in %): relative volume of the structure exposed to at least 2 or 5 Gy, respectively. LMCA: left main common coronary artery, LAD: left anterior descending artery, CX: circumflex artery, RC: right coronary artery, ^1^ based on Wilcoxon test.

**Table 3:** Comparison of CAC (overall and by coronary artery) before RT and two years after RT.

CAC Description	All Patients *N* = 101			Right-Sided BC *N* = 17			Left-Sided BC *N* = 84		
	Before RT	RT+ Two Years	*p*-Value	Before RT	RT+ Two Years	*p*-Value	Before RT	RT+ Two Years	*p*-Value
**Overall CAC**									
Non-zero CAC, *N* (%)	25 (24.7%)	30 (29.7%)	0.062	3 (17.6%)	3 (17.6%)	1.000 ^1^	22 (26.2%)	27 (32.1%)	0.062 ^1^
Median/Mean (± SD)	0/35.8 (± 119.1)	0/47.7 (± 140.0)	<0.001	0/3.2 (± 11.1)	0/4.5 (± 14.9)	0.158 ^2^	0/42.4 (± 129.5)	0/56.4 (± 151.9)	<0.001 ^2^
*CAC* progression, N (%)	28 (27.7%)			2 (11.7%)			26 (30.1%)		
**Coronary artery-specific CAC**									
**LMCA CAC**									
Non-zero CAC, N (%)	2 (2.0%)	2 (2.0%)	1.000	0	0	1.000 ^1^	2 (2.4%)	2 (2.4%)	1.000 ^1^
Median/Mean (± SD)	0/0.3 (±2.8)	0/ 0.5 (±4.9)	0.157	0	0	1.000 ^2^	0/ 0.4 (±3.1)	0/ 0.6 (±5.4)	0.157 ^2^
Increased CAC, N (%)	2 (2.0%)			0			2 (2.4%)		
**LAD CAC**									
Non-zero CAC, N (%)	20 (19.8%)	26 (25.7%)	0.031	1 (5.9%)	2 (11.8%)	1.000 ^1^	19 (22.6%)	24 (28.5%)	0.062 ^1^
Median/Mean (± SD)	0/20.6 (±58.9)	0/ 27.6 (±74.3)	<0.001	0/ 0.3 (±1.5)	0/ 1.0 (±3.4)	0.158 ^2^	0/ 24.7 (±63.9)	0/ 33.1 (±80.5)	<0.001 ^2^
Increased CAC, N (%)	26 (25.7%)			2 (11.8%)			24 (28.6%)		
**CX CAC**									
Non-zero CAC, N (%)	7 (6.9%)	10 (9.9%)	0.250	0	0	1.000 ^1^	7 (8.3%)	10 (11.9%)	0.250 ^1^
Median/Mean (± SD)	0/3.7 (±23.5)	0/ 5.2 (±26.3)	0.016	0	0	1.000 ^2^	0/4.48±25.80	0/6.2 (±28.8)	0.016 ^2^
Increased CAC, N (%)	10 (9.9%)			0			10 (11.9%)		
**RCA CAC**									
Non-zero CAC, N (%)	15 (14.8%)	17 (16.8%)	0.500	2 (11.7%)	2 (11.7%)	1.000 ^1^	13 (15.4%)	15 (17.8%)	0.500 ^1^
Median/Mean ± SD	0/11.2 (±55.2)	0/16.3 (±68.9)	<0.001	0/2.8 (±11.1)	0/3.7 (±14.7)	0.158 ^2^	0/12.8(±60.3)	0/18.8 (±75.1)	<0.001 ^2^
Increased CAC, N (%)	16 (16.7%)			2 (11.7%)			14 (16.7%)		

CAC: coronary artery calcium score, LMCA: left main common coronary artery, LAD: left anterior descending artery, CX: circumflex artery; RCA right coronary artery. CAC progression: patients with CAC_RT+2 years_–CAC_Before RT_ > 0; ^1^ based on Mac Nemar test: ^2^ based on Wilcoxon test for paired samples.

**Table 4:** Association between CAC progression (yes/no) and non-radiation cardiovascular risk factors.

Variables	OR (95% CI)	*p*-Value
Age, in years	1.09 (1.03–1.16)	0.005
BMI, in kg/m²	1.09 (0.98–1.21)	0.107
arterial hypertension	3.71 (1.20–11.51)	0.023
Diabetes	7.72 (1.40–42.50)	0.019
Smoking (former or current)	2.15 (0.76–6.11)	0.151
Hypercholesterolemia	1.86 (0.75–4.59)	0.179
Cardiovascular treatment	5.29 (1.67–16.73)	0.005
Baseline CAC score > 0	163.3 (29.6–899.0)	<0.0001
Endocrine therapy	0.55 (0.21–1.45)	0.224

**Table 5:** Association between total CAC progression (yes/no) and cardiac exposure.

Exposure Variables	Univariate Model		Multivariable Model 1		Multivariable Model 2	
	OR (95% CI)	*p*-Value	OR (95% CI)	*p*-Value	OR (95% CI)	*p*-Value
**Left vs. Right-sided BC**	3.36 (0.72–15.78)	0.12	3.22 (0.61–16.80)	0.16	16.55 (0.091–302.26)	0.08
**Heart**						
Mean dose, in Gy	1.32 (0.97–1.80)	0.07	1.29 (0.91–1.84)	0.15	1.74 (0.94–3.21)	0.08
D2, in Gy	1.03 (1.00–1.06)	0.02	1.02 (0.99–1.05)	0.13	1.03 (0.98–1.07)	0.23
V2, in %	1.03 (0.99–1.05)	0.10	1.01 (0.98–1.05)	0.43	1.07 (1.01–1.12)	0.01
V5, in %	1.04 (0.97–1.11)	0.28	1.05 (0.97–1.14)	0.25	1.11 (0.98–1.26)	0.08
**Left Ventricle**						
Mean dose, in Gy	1.20 (1.06–1.37)	0.01	1.15 (1.00–1.32)	0.04	1.29 (1.01–1.65)	0.04
D2, in Gy	1.05 (1.02–1.08)	0.01	1.04 (1.01–1.07)	0.02	1.07 (1.01–1.14)	0.03
V2, in %	1.03 (1.01–1.06)	0.01	1.03 (0.99–1.06)	0.05	1.07 (1.01–1.12)	0.01
V5, in %	1.05 (1.01–1.09)	0.02	1.04 (0.99–1.08)	0.09	1.01 (1.01–1.19)	0.02

Model 1: adjusted on age, hypertension, diabetes, and cardiovascular treatment Model 2: adjusted on baseline CAC status (zero/non-zero) and age.

**Table 6:** Association between LAD CAC progression (yes/no) and cardiac exposure measurements.

Exposure Variables	Univariate Model		Multivariable Model *	
	OR (95% CI)	*p*-Value	OR (95% CI)	*p*-Value
**Left vs. Right-sided BC**	3.00 (0.63–14.1)	0.16	2.75 (0.54–14.11)	0.22
**Heart**				
Mean dose, in Gy	1.24 (0.91–1.69)	0.17	1.18 (0.82–1.70)	0.36
D2, in Gy	1.03 (1.00–1.05)	0.06	1.01 (0.99–1.04)	0.34
V2, in %	1.02 (0.99–1.05)	0.15	1.01 (0.98–1.05)	0.54
V5, in Gy	1.03 (0.96–1.10)	0.42	1.03 (0.95–1.12)	0.43
**Left Ventricle**				
Mean dose, in Gy	1.20 (1.05–1.36)	0.01	1.14 (0.99–1.31)	0.06
D2, in Gy	1.04(1.01–1.07)	0.01	1.03 (1.00–1.06)	0.06
V2, in %	1.04 (1.01–1.06)	0.01	1.03 (1.00–1.06)	0.04
V5, in Gy	1.05 (1.01–1.09)	0.02	1.04 (0.99–1.09)	0.09
**LAD**				
Mean dose, in Gy	1.03 (0.98–1.09)	0.20	1.04 (0.99–1.10)	0.14
D2, in Gy	1.04 (1.01–1.07)	0.02	1.03 (1.00–1.06)	0.07
V2, in %	1.02 (1.00–1.03)	0.08	1.02 (1.00–1.04)	0.08
V5, in Gy	1.01 (0.99–1.03)	0.31	1.01 (0.99–1.03)	0.21

* model 1 adjusted for age, diabetes, and cardiovascular treatments.

## Data Availability

The datasets used and/or analyzed during the current study are available from the corresponding author on reasonable request.
